# Rsp promotes the transcription of virulence factors in an *agr*-independent manner in *Staphylococcus aureus*

**DOI:** 10.1080/22221751.2020.1752116

**Published:** 2020-04-24

**Authors:** Banghui Liu, Baolin Sun

**Affiliations:** Department of Oncology, The First Affiliated Hospital, University of Science and Technology of China, Hefei, People’s Republic of China

**Keywords:** *Staphylococcus aureus*, Rsp, Agr, haemolysis, Hla, PSMs, virulence

## Abstract

*Staphylococcus aureus* is a major human pathogen that causes a great diversity of community- and hospital-acquired infections. Rsp, a member of AraC/XylS family of transcriptional regulators (AFTRs), has been reported to play an important role in the regulation of virulence determinants in *S. aureus* via an *agr*-dependent pathway. Here we demonstrated that Rsp could bind to the *rsp* promoter to positively regulate its own expression. We then constructed an isogenic *rsp* deletion strain and compared the haemolysis in the wild-type and *rsp* mutant strains. Our results indicated that the *rsp* mutant strain displayed decreased haemolytic activity, which was correlated with a dramatic decrease in the expression of *hla* and *psm*. Furthermore, we analysed the regulatory effects of Rsp in the *agr* mutant strain and found that they are *agr*-independent. Electrophoretic mobility shift assay indicated that Rsp can directly bind to the promoter regions of *hla* and *psm*. The mouse model of subcutaneous abscess showed that the *rsp* mutant strain displayed a significant defect in virulence compared to the wild-type strain. These findings reveal that Rsp positively regulates the virulence of *S. aureus* by promoting the expression of *hla* and *psm* through direct binding to their promoter regions.

## Introduction

*Staphylococcus aureus* is a detrimental and versatile human pathogen responsible for a wide diversity of the community- and hospital-acquired infections, ranging from innocuous skin infections to life-threatening conditions like pneumonia, osteomyelitis and infective endocarditis [1,2]. The pathogenicity of the bacterium is a sophisticated process involving multiple virulence determinants, such as exotoxins, enzymes, and surface protein adhesins [3,4]. The expression of virulence factors is coordinately controlled by three global regulators, *agr*, *sarA*, and *sae* [5], which are believed to enable *S. aureus* to survive and to elicit subsequent infection in different environments.

The AraC/XylS family [6] is a group of transcriptional regulators with a highly conserved 99 amino acid at the C-terminal region. Members of this family are widely distributed in numerous species of gram-negative and gram-positive bacteria and mainly involved in metabolism of carbon sources, responses to environmental stress and pathogenesis. In *S. aureus*, 6 AraC/XylS-like proteins are predicted [7] and four of which, Rbf, Rsp, AryK and HptR, have been experimentally characterized. Rbf was initially reported to participate in biofilm formation in response to sodium chloride and glucose [8], and subsequent studies demonstrated that Rbf can promote biofilm formation via repression of *icaR* [9] and activation of *sarX* [10]. Rsp originally was described to modulate biofilm formation by repressing surface proteins [11], and further studies showed that Rsp was essential for the expression of virulence factors and the development of pneumonia and skin infections in mouse models [12,13]. AryK was shown to potentiate toxin expression and virulence of *S. aureus* JDK6159, a highly virulent strain, by a loss-of-function point mutation [14]. HptR, a response regulator protein of three-component regulatory system HptRSA, was found to facilitate the uptake of glucose-6-phosphate (G6P) in *S. aureus* and support the bacterial survival and proliferation in host cells [15,16]. Therefore, the regulatory effects of the AraC/XylS family proteins in *S. aureus* are varied, and much of them still remains to be explored.

The *agr* locus is the most investigated quorum-sensing system of staphylococci and consists of two divergent transcripts, RNAII and RNAIII, driven by P2 and P3 promoters, respectively [17]. The RNAII transcript encodes a typical two-component signal-transduction system, which comprises the sensor histidine kinase AgrC and the response regulator AgrA in response to the extracellular concentration of the autoinducing peptide (AIP) encoded and modified by the proteins AgrD and AgrB [18,19]. Induction of *agr* results in the amplification of quorum-sensing signal and the expression of 514-nucleotide transcript RNAIII, the major effector of the *agr* system, mediating the expression of *agr* regulon by an antisense mechanism [20]. The *agr* system is critical for the pathogenicity of *S. aureus* and can modulate the expression of virulence factors in both RNAIII-dependent and RNAIII-independent patterns. In the RNAIII-independent manner, the *agr* system regulates the transcription of virulence genes by direct binding of AgrA to the promoters of target genes [21].

Hla and PSMs are the two prominent and well-characterized cytotoxins in *S. aureus*, and both of them are pore-forming toxins. Hla is a hydrophilic polypeptide that presents in culture supernatant as a monomer [22]. Oligomerization into hexamers or heptamers on the host membrane mediated by its cellular receptor ADAM10 triggers the pore formation and the membrane lysis [23]. Thus, with the ability to lyse a wide array of cell types, this toxin has been recognized as an important contributor to the pathogenesis of pneumonia, sepsis, mastitis, and skin infections [24–28]. PSMs are small, amphipathic peptides consisting of five shorter α-type peptides (PSMα1-PSMα4 and δ-toxin) and two longer β-type peptides (PSMβ1-PSMβ2) [29]. PSMαs are transcribed from the *psmα* operon, PSMβs are transcribed from the *psmβ* operon, and the δ-toxin is transcribed from the agrP3 promoter [29,30]. Unlike Hla, the ability of PSMs to lyse eukaryotic cells is receptor-independent [31]. Among the 7 peptides produced by *S. aureus*, the PSMα peptides, especially PSMα3 showed by far the strongest cytolytic activity [32]. Deletion of the *psmα* operon in community-associated methicillin-resistant *S. aureus* (CA-MRSA) significantly decreases the ability to cause skin and soft-tissue infections in mice and the capacities to attract and lyse neutrophils [33,34]. Compared with hospital-associated (HA)-MRSA, the CA-MRSA shows much higher *in vitro* expression of PSMs, giving the fact that PSMs peptides contribute to a great extent to the enhanced virulence of CA-MRSA [29,35]. The expression of *hla* and *psm* is strictly regulated by global virulence regulators. *hla* is tightly controlled by the positive regulators sRNA RNAIII, SarA and Sae [36–38], and the negative regulators Rot and SarT [39,40]. The expression of *psm* is positively regulated by the *agr* system through the direct binding of AgrA to the promoter regions of *psm* operons [21]. Although the functions and regulations of *hla* and *psm* have been studied extensively, it is necessary to identify the potential transcriptional regulators of *hla* and *psm* to provide a greater insight into pathogenic mechanisms.

Previously, Rsp has been reported to regulate the expression of virulence genes via an *agr*-dependent pathway [12]. However, whether Rsp directly regulates the expression of these genes remains unknown. In this study, we demonstrated that Rsp could bind to the *rsp* promoter to upregulate its own expression. Additionally, we found that Rsp can positively regulate the expression of *hla*, *psmα*, and *psmβ* by directly binding to their promoter regions in an *agr*-independent manner. The contribution of Rsp to the pathogenicity of *S. aureus* was further confirmed by using a mouse subcutaneous abscess model.

## Materials and methods

### Bacterial strains, plasmids, and growth conditions

The bacterial strains and plasmids used in this study are listed in Table 1. *Escherichia coli* strains were cultivated with shaking (220 rpm) in lysogeny broth (LB) medium (Oxoid) or on lysogeny broth agar (LA) at 37°C. *S. aureus* strains were grown with shaking (220 rpm) in tryptic soy broth (TSB) medium (Difco) or on tryptic soy agar (TSA) at 37°C. When required, appropriate antibiotics were used for plasmid selection and maintenance at the following concentrations: for *E. coli*, ampicillin at 150 μg/ml and kanamycin at 50 μg/ml; for *S. aureus*, chloramphenicol at 15 μg/ml.

**Table 1.** Strains and plasmids used in this study.

Strain or plasmid	Relevant genotype^a^	Reference or source
*S. aureus* strains		
RN4220	8325-4 r^-^, initial recipient for modification of plasmids which are introduced into *S. aureus* from *E. coli*	NARSA^b^
NCTC8325	HA-MSSA, wild-type	NARSA
Δ*rsp*	8325 strain deletion of *rsp*	This study
C*rsp*	*rsp* chromosomal complementation of Δ*rsp*	This study
Δ*agr*	8325 strain deletion of *agr*	This study
Δ*agr*Δ*rsp*	8325 *agr rsp* double mutant	This study
N315	HA-MRSA, *agr*-deficient	NARSA
N315Δ*rsp*	N315 strain deletion of *rsp*	This study
Newman	HA-MSSA, *α*- and *β*-toxin-deficient	[41,42]
NewmanΔ*rsp*	Newman strain deletion of *rsp*	This study
*E. coli* strains		
Trans1-T1	Clone host strain, F^-^ ϕ80 (*lacZ*) ΔM15Δ*lacX74 hsdR* (r_K_^-^ m_K_^+^) Δ*recA*1398 *endA1 tonA*	TransGen
BL21(DE3)	Express strain, F^-^ *ompT hsdS_B_* (r_B_^-^ m_B_^-^) *gal dcm* (DE3)	TransGen
Plasmids		
pBTs	Shuttle vector, temp sensitive, amp^r^ cm^r^	[43]
pBTsΔ*rsp*	pBTs derivative, for *rsp* deletion, amp^r^ cm^r^	This study
pBTs-up-*rsp*-down	pBTs derivative, for *rsp* chromosomal complementation, amp^r^ cm^r^	This study
pBTsΔ*agr*	pBTs derivative, for *agr* deletion, amp^r^ cm^r^	This study
pLI50	Shuttle vector, amp^r^ cm^r^	[44]
pLI*rsp*	pLI50 derivative, harbouring ORF of *rsp* and its promoter, amp^r^ cm^r^	This study
pRMC2	Shuttle vector, anhydrspetracycline inducible, amp^r^ cm^r^	[45]
pRMC*rsp*	pRMC2 derivative, harbouring ORF of *rsp*, amp^r^ cm^r^	This study
pOS1-*lacZ*	Shuttle vector, with *lacZ* ORF lacking first 6 amino acids, amp^r^ cm^r^	[46]
pOS*rsp*	POS1-*lacZ* derivative, harbouring 301-bp region of *rsp* promoter and 18 bp of *rsp* coding sequence from strain NCTC8325, amp^r^ cm^r^	This study
pOS*hla*	POS1-*lacZ* derivative, harbouring 235-bp region of *hla* promoter and 18 bp of *hla* coding sequence from strain NCTC8325, amp^r^ cm^r^	[47]
pOS*psmα*	POS1-*lacZ* derivative, harbouring 308-bp region of *psmα* promoter and 18 bp of *psmα* coding sequence from strain NCTC8325, amp^r^ cm^r^	This study
pOS*psmβ*	POS1-*lacZ* derivative, harbouring 226-bp region of *psmβ* promoter and 18 bp of *psmβ* coding sequence from strain NCTC8325, amp^r^ cm^r^	This study
pRSF-Duet	Expression vector with a hexa-histidine tag, kan^r^	Novagen
pRSF-Duet-Rsp	pRSF-Duet derivative, with ORF of *rsp*, kan^r^	This study

^a^r^−^, restriction system negative; kan^r^, kanamycin resistant; amp^r^, ampicillin resistant; cm^r^, chloramphenicol resistant.

^b^NARSA, Network on Antimicrobial Resistance in *Staphylococcus aureus*.

### Construction of the rsp mutant, agr mutant, and agr rsp double mutant strains

To construct the *rsp* mutant strain, the plasmid pBTs was used as described previously [43]. The upstream and downstream regions of *rsp*, which were amplified from genomic DNA of *S. aureus* strains NCTC8325, N315, and Newman, respectively, using primer pairs *rsp*-up-F/*rsp*-up-R and *rsp*-down-F/*rsp*-down-R. The products were ligated by overlap PCR to form an up–down fragment. The resultant fragment was digested with KpnI and SalI, and cloned into the KpnI/SalI-digested plasmid pBTs, which containing a temperature-sensitive *S. aureus* origin of replication, a chloramphenicol resistance cassette, and a suicide gene for plasmid maintenance or selection. The resulting plasmid, pBTsΔ*rsp*, was first electroporated into *S. aureus* strain RN4220 for modification and subsequently transformed into *S. aureus* strains NCTC8325, N315, and Newman. An allelic replacement mutant was selected using a previously described method [48] and was further confirmed by PCR and sequencing. The *agr* mutant and *agr rsp* double mutant strains were constructed using a similar strategy by introducing the plasmid pBTsΔ*agr* into the wild-type (WT) and *rsp* mutant strains, respectively. All primers used in this study are listed in Table 2.

**Table 2.** Primers used in this study.

Primer	Sequence (5′–3′)^a^	Application
*rsp*-up-F-KpnI	GCGggtaccAAGGTGTTAATTCATTGATG	*rsp* deletion
*rsp*-up-R	ATTTCTCTCTCCTGCCTAAT	*rsp* deletion
*rsp*-down-F	**ATTAGGCAGGAGAGAGAAAT**ATACTAACAGTCCTCTTGTG	*rsp* deletion
*rsp*-down-R-SalI	ACGCgtcgacAACTAATACCAAGACCAAAA	*rsp* deletion
C*rsp*-F-KpnI	GCGggtaccTTACGGCAATAACTAGATGG	*rsp* chromosomal complementation
C*rsp*-R-SalI	ACGCgtcgacTGCAGTATTTATATTACATA	*rsp* chromosomal complementation
*agr*-up-F-KpnI	GCGggtaccAAGAGGTTGAACAAGCATTT	*agr* deletion
*agr*-up-R	GCAGCGATGGATTTTATTTT	*agr* deletion
*agr*-down-F	**AAAATAAAATCCATCGCTGC**GATGAATAATTAATTACTTTCAT	*agr* deletion
*agr*-down-R-SalI	ACGCgtcgacGTCATTGGAACTAATAGCAC	*agr* deletion
pLI50-*rsp*-F-SacI	TCCgagctcTATTGTTTTTTGAAATACATAGG	*rsp* overexpression
pLI50-*rsp*-R-KpnI	CGGggtaccTTGAATTGCTTGTGAGTTAC	*rsp* overexpression
pRMC2-*rsp*-F-KpnI	CGGggtaccATTAGGCAGGAGAGAGAAAT	*rsp* induced expression
pRMC2-*rsp*-R-EcoRI	CCGgaattcAGGACTGTTAGTATTTAGCT	*rsp* induced expression
RT-*rsp*-F	ACGATATTCACGATATTGAGATT	qRT-PCR
RT-*rsp*-R	TCTTGCTTGTCTTATAGTCTTG	qRT-PCR
RT-*agrA*-F	TATGGCGATTGACGACAA	qRT-PCR
RT-*agrA*-R	GCAGTAATTCAGTGTATGTTCA	qRT-PCR
RT-*psmα*-F	GTATCATCGCTGGCATCA	qRT-PCR
RT-*psmα*-R	AAGACCTCCTTTGTTTGTTATG	qRT-PCR
RT-*psmβ*-F	TGGACTAGCAGAAGCAATC	qRT-PCR
RT-*psmβ*-R	TAGTAAACCCACACCGTTAG	qRT-PCR
RT-*hla*-F	GGTATATGGCAATCAACTT	qRT-PCR
RT-*hla*-R	CTCGTTCGTATATTACATCTAT	qRT-PCR
RT-*hu*-F	AAAAAGAAGCTGGTTCAGCAGTAG	qRT-PCR
RT-*hu*-R	TTTACGTGCAGCACGTTCAC	qRT-PCR
P*rsp*-F-EcoRI	CCGgaattcGTATTGTTTTTTGAAATACATAG	pOS*rsp*
P*rsp*-R-BamHI	CGCggatccGGTTTAAGTTGGCATGTCATAT	pOS*rsp*
P*hla*-F-EcoRI	CCGgaattcTTCAACTTTGACTAACCCTC	pOS*hla*
P*hla*-R-BamHI	CGCggatccGGGACTATACGTGTTTTCATTT	pOS*hla*
P*psmα*-F-EcoRI	CCGgaattcTCTGTTCAATTCATCTTCATA	pOS*psmα*
P*psmα*-R-BamHI	CGCggatccGGGCCAGCGATGATACCCATTAAG	pOS*psmα*
P*psmβ*-F-EcoRI	CCGgaattcGGCAACTTAATTGTGTTAAA	pOS*psmβ*
P*psmβ*-R-BamHI	CGCggatccGGGTTAAATAAACCTTCCATTG	pOS*psmβ*
E-*rsp*-F-BamHI	CGCggatccGATGACATGCCAACTTAAAAT	Rsp expression
E-*rsp*-R-EcoRI	CCGgaattcTTAGCTTGGTTTAAAGCAAA	Rsp expression
probe-p*rsp*-F319	GTATTGTTTTTTGAAATACATAG	EMSA
probe-p*rsp*-F139	TTCACTATTACCTTTTCAAAA	EMSA
probe-p*rsp*-F89	TGATTTTAAAAATTTCCATG	EMSA
probe-p*rsp*-Biotin-R	TTTAAGTTGGCATGTCATAT	EMSA
probe-p*hla*-F310	TTAGCTATGTCTTTTCCTTG	EMSA
probe-p*hla*-F239	CCTTTCAAATTTTAAATAAA	EMSA
probe-p*hla*-F189	CATCATCACTCAGTAATTTA	EMSA
probe-p*hla*-F110	TGCAATATTCTAAATTGACA	EMSA
probe-p*hla*-Biotin-R	TAGTGTTGTTGTTACTGAG	EMSA
probe-p*psmα*-F326	TCTGTTCAATTCATCTTCATA	EMSA
probe-p*psmα*-F166	ACCTGTACAGTTAGGCAGTA	EMSA
probe-p*psmα*-F85	AAAATCAATTACGCACAAGA	EMSA
probe-p*psmα*-Biotin-R	GCCAGCGATGATACCCATTAAG	EMSA
probe-p*psmβ*-F253	ACTTAAATACGAATTCAGGCAACT	EMSA
probe-p*psmβ*-F162	CATTATAAGATGTTGTGCGG	EMSA
probe-p*psmβ*-F139	AACAAACTAATTGCATCAAA	EMSA
probe-p*psmβ*-F89	AAGCGAATAACATTTCGGTG	EMSA
probe-p*psmβ*-Biotin-R	AACCTTCCATTGAAAACACTCC	EMSA

^a^Lowercase letters indicate restriction sites. Letters in bold indicate complementary sequences used for overlap pcr ligation.

### Construction of the complementation and overexpression strains

For *rsp* chromosomal complementation, the DNA fragment covering the open reading frame (ORF) of *rsp* and the flank upstream and downstream regions was amplified from *S. aureus* strain NCTC8325 genomic DNA using the primer pair C*rsp*-F/C*rsp*-R. The PCR product was digested with KpnI and SalI, and then cloned into the pBTs vector. The resulting plasmid, pBTs-up-*rsp*-down, was first electroporated into *S. aureus* strain RN4220 for modification and subsequently transformed into the *rsp* mutant strain. The allelic replacement complementation strain was selected using the same method described above and was further confirmed by PCR and sequencing.

For *rsp* overexpression, both the plasmid pLI*rsp* and pRMC*rsp* were employed. To construct pLI*rsp*, the fragment encompassing the ORF of *rsp* and its native promoter was amplified from *S. aureus* strain NCTC8325 genomic DNA using the primer pair pLI50-*rsp*-F/pLI50-*rsp*-R. The fragment was digested with SacI and KpnI, and then cloned into the shuttle plasmid pLI50 to derive the plasmid pLI*rsp*. To construct pRMC*rsp*, the fragment encompassing the ORF of *rsp* was amplified from *S. aureus* strain NCTC8325 genomic DNA using the primer pair pRMC-*rsp*-F/pRMC-*rsp*-R. The fragment was digested with KpnI and EcoRI, and then cloned into the shuttle plasmid pRMC2 with an anhydrotetracycline (ATC) inducible promoter. The combinant plasmids were first electroporated into *S. aureus* strain RN4220 for modification and subsequently transformed into the WT and *agr* mutant strains to derive the overexpressed strains.

### Total RNA isolation, cDNA generation, and real-time quantitative reverse transcription-PCR

Overnight cultures of *S. aureus* were diluted 1:100 in TSB and then grown to the indicated cell density until being collected. *S. aureus* cells were collected by centrifugation and processed with 1 ml of RNAiso plus (TaKaRa) in combination with 0.1-mm-diameter silica-zirconia beads in a FastPrep-24 automated system (MP Biomedicals), and the residual DNA was removed with RNase-free DNase I (TransGen, 3 U/μl). The concentration of total RNA was adjusted to 200 ng/μl. For the reverse transcription, the cDNAs were synthesized with a PrimeScript 1st Strand cDNA synthesis kit (TaKaRa) using random primers and real-time quantitative reverse transcription-PCR (qRT-PCR) was performed with SYBR Premix *Ex Taq* (TaKaRa) using the StepOne Real-Time PCR System (Applied Biosystems) and LC96 Real-Time PCR System (Roche). The quantity of cDNA measured by real-time PCR was normalized to the abundance of *hu* cDNA [49], and the corresponding control sample as the run calibrator. All qRT-PCR assays were repeated at least three times.

### Determination of haemolytic activity

Qualitative evaluation of haemolytic activity was evaluated on sheep blood agar (SBA). The OD_600_s of overnight culture were measured and adjusted to the same level. Two-microliter culture volumes were then spotted onto SBA plates. The plates were left to dry for about 2 min and incubated at 37°C for 24 h, and visually evaluated for zones of lysis.

Quantitative evaluation of haemolytic activity was determined by incubating samples with sheep red blood cells. Overnight cultures were collected by centrifugation, and supernatants (100 μl) were mixed with 900 μl phosphate-buffered saline (PBS) buffer containing 3% sheep red blood cells, and the mixtures were incubated at 37°C for proper time. The absorption of supernatant at 543 nm was measured after centrifugation. A mixture with 1000 μl ddH_2_O containing 3% sheep red blood cells was used as the positive control, and a mixture with 1000 μl PBS containing 3% sheep red blood cells was used as the negative control. The percentage of haemolytic activity was calculated relative to the positive control, which was regarded as 100% haemolytic activity. For hemolysin-deficient strains, the supernatants were mixed with PBS containing 15% sheep red blood cells. A mixtures with 1000 μl ddH_2_O and 1000 μl PBS containing 15% sheep red blood cells were used as the positive control and the negative control, respectively.

### Expression and purification of Rsp

The 6-His-tagged Rsp was expressed and purified using standard procedures. The fragment of the full-length *rsp* ORF was amplified by PCR with the primer pair E-*rsp*-F/E-*rsp*-R from *S. aureus* strain NCTC8325 genomic DNA, cloned into the expression vector pRSF-Duet to generate the plasmid pRSF-Duet-Rsp, and transformed into *E. coli* BL21 (DE3). The transformant was grown in LB at 37°C to an OD_600_ of 0.6 and induced with 0.5 mM isopropyl-β-D-1-thiogalactopyranoside (IPTG) at 16°C for additional 12 h. The cells were harvested and lysed by sonication in a lysis buffer (50 mM Tris-HCl, pH 8.0, 300 mM NaCl). The hexahistidine-tagged Rsp protein was purified with a nickel-nitrilotriacetic acid-agarose solution (Qiagen) by following the manufacturer’s recommendation. The bound protein was eluted with an elution buffer (200 mM imidazole, 50 mM Tris-HCl, pH 8.0, 300 mM NaCl). The imidazole in the eluent was removed using a Centrifuge Biomax-5 column (Millipore). The purity of the protein was analysed using SDS-PAGE, and the protein concentration was determined using the bicinchoninic acid (BCA) assay with bovine serum albumin as the standard.

### Electrophoretic mobility shift assay

The biotin-labelled DNA fragments containing the putative promoter regions of *rsp* (319 bp), which was predicted using BPROM (http://linux1.softberry.com/berry.phtml) and *hla* (310 bp), *psmα* (326 bp) or *psmβ* (253 bp), which were based on our previous studies [47,50], were amplified from *S. aureus* strain NCTC8325 genomic DNA. The biotin-labelled probe was incubated at 25°C for 30 min with various amounts of Rsp in 10 μl of incubation buffer (50 mM Tris–HCl, pH 8.0, 300 mM NaCl). After incubation, the mixtures were electrophoresed in a 5% native polyacrylamide gel in 1 × Tris-borate-EDTA (TBE) buffer and then transferred to a nylon membrane in 0.5 × TBE buffer. The band shifts were detected using the Chemiluminescent Nucleic Acid Detection Module Kit (ThermoFisher), and imaged with the ImageQuant LAS 4000 (GE Healthcare). The unlabelled fragments of each promoter were added to the labelled fragments at a ratio of approximately 100:1 or 200:1 as specific competitors. The unlabelled DNA fragment of the *hu* ORF (125 bp) (150-fold or 200-fold) was added as a nonspecific competitor.

### Construction of the LacZ reporter vector

To construct the reporter plasmid pOS*rsp* for detection of *rsp* expression, a fragment containing the 301-bp promoter of *rsp* and the first 18-bp region of the *rsp* coding sequence was amplified from *S. aureus* strain NCTC8325 genomic DNA with primer pair P*rsp*-F/R. The fragment was digested with BamHI and EcoRI and cloned into the shuttle vector pOS1-*lacZ* to generate the reporter plasmid pOS*rsp*. The same protocol was followed to construct the reporter plasmids pOS*hla,* pOS*psmα* and pOS*psmβ* using primer pairs P*hla*-F/R, P*psmα*-F/R and P*psmβ*-F/R, respectively. These reporter plasmids were first transformed into *S. aureus* strain RN4220, and then the WT and *rsp* mutant strains.

### β-Galactosidase activity assay

For β-galactosidase assay with o-nitrophenyl-β-D-galactopyranoside (ONPG) as the substrate, overnight cultures of the WT and *rsp* mutant strains containing different reporter plasmids were diluted 1:100 into TSB with chloromycetin (15 μg/ml) and grown to the indicated cell density until being collected. The collected cells were resuspended in 100 μl of ABT-LSA buffer (60 mM K_2_HPO_4_, 40 mM KH_2_PO_4_, 100 mM NaCl, 0.1% Triton X-100, 50 μg/ml lysostaphin) and incubated at 37°C until thoroughly lysed. Then, 100 μl ABT buffer and 100 μl 4 mg/ml ONPG were added to initiate the reaction. After incubation at 37°C for the proper time (0–1 h), the reactions were terminated by the addition of l ml Na_2_CO_3_ (1 M). Sample absorbance was read at 420 nm and units were calculated as the following formula: units = (1000 × OD_420_)/(*T* × *V* × OD_600_). *T* (measured in minutes) was the incubation time and *V* (in millilitres) was the volume of culture used in the assay.

### mRNA half-lives

For mRNA half-life determination, overnight culture of *S. aureus* was inoculated at 1:100 into fresh medium and grown to mid-exponential, transcription was arrested by the addition of rifampin (200 µg/ml), and aliquots were removed at 0-, 3-, 5-, 10-, 15-, 30- and 60- min post-transcriptional arrest. RNA was isolated from each aliquot and the mRNA levels of p*rsp*-*lacZ* and *rsp* were measured by qRT-PCR.

### Western blot analysis

To detect the production of Hla, stationary-phase culture supernatant was collected and heated for 10 min at 95°C. The samples were then separated by 12% SDS-PAGE and electrotransferred onto a polyvinylidene difluoride membrane (GE, Piscataway, NJ). After blocking with 5% (w/v) nonfat milk in TBST buffer at room temperature for 1 h, the membrane was incubated with a rabbit anti-Hla IgG antibody (Sigma) at a 1/2500 dilution. Bound antibody was detected with the goat anti-rabbit conjugated to horseradish peroxidase (ThermoFisher) at a 1/5000 dilution. The images were obtained with ImageQuant LAS 4000 (GE Healthcare).

### Mouse skin infection model

Outbred, immunocompetent female BALB/c mice with age of 6 weeks were purchased from Beijing Vital River Laboratory Animal Technology Company, and housed in isolated cages in an animal facility under specific pathogen-free. The hair on the back was removed by an animal shaver. Overnight cultures were diluted 1:100 into TSB and grown to the indicated cell density until being collected. The collected cells were washed twice and diluted in sterile PBS, and viable cells were counted via their colony-forming units (CFU) on TSA plates in order to quantify the infectious dose. Mice were anesthetized with 1% pentobarbital sodium and inoculated by subcutaneous injection in both flanks of the back with 5 × 10^7^ live *S. aureus* cells in 50 μl PBS. Abscess formation and skin lesion area were monitored at 24-h intervals for 7 days. The sizes of the skin lesions were calculated by the maximal length × width, as previously described [51]. The skin lesions were excised 7 days after infection and homogenized in PBS. The CFU recovered from each individual lesion was determined by serial dilution and plated onto TSA plates.

### Biofilm formation and analysis

Biofilm formation under static conditions was determined by the microtiter plate assay based on the method described previously [52]. Briefly, overnight cultures were diluted 1:200 in fresh TSB medium and dispensed into wells of sterile flat-bottom 96-well polystyrene plates (Costar) at 200 μl per well. The plates were incubated at 37°C for 24 h, and the wells were washed gently three times with water to remove non-adherent cells. The plates were stained with 0.5% crystal violet for 20 min, and then again washed three times with water. After that, the plates were dried, and the optical density at 560 (OD_560_) was determined with an enzyme-linked immunosorbent assay reader ELX800 (Bio-Tek) in a 3 × 3 scan model. To investigate the biofilm formation of the *S. aureus* strains N315 and Newman, the TSB medium was supplemented with 0.25% glucose.

### Ethics statement

The use and care of mice in the present study followed strictly the guidelines adopted by the Ministry of Health of the People’s Republic of China in June 2004. The protocol was approved by the Institutional Animal Care and Use Committee of the University of Science and Technology of China (USTCACUC192001037).

### Statistical analyses

*F* tests of two samples for variance were performed. Unpaired two-tailed *t* tests for equal or unequal variance were then performed to calculate the significant differences (*P* values). A value of *P* < .05 was considered statistically significant.

## Results

### Rsp promotes the transcription of rsp through a self-activation manner

**Figure 1.**
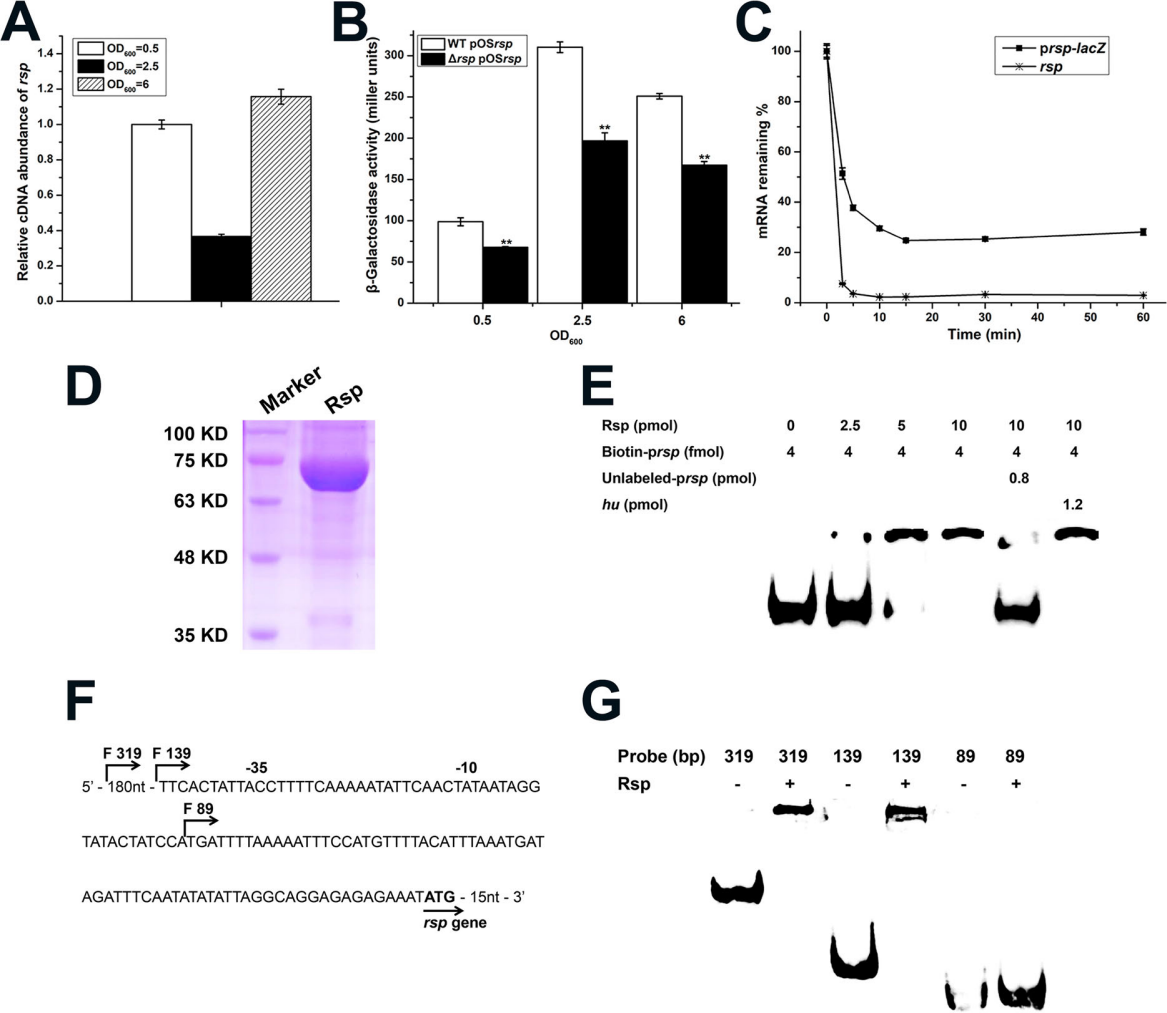
Rsp positively regulates its own expression. (A) The transcript levels of *rsp* at different growth phases were measured by qRT-PCR. (B) The β-galactosidase activities of the WT and *rsp* mutant strains with a plasmid pOS*rsp* were detected in the indicated time points. (C) Analysis of mRNA half-lives of p*rsp*-*lacZ* and *rsp*. Cells were collected after rifampin (200 μg/ml) treatment for RNA isolation, and then the mRNA half-lives were measured by qRT-PCR. (D) SDS-PAGE analysis of Rsp purified from the pRSF-Duet expression vector. The gel was stained with Coomassie blue. (E) EMSA of purified Rsp with the biotin-labelled DNA fragment containing the putative promoter region of *rsp*. Increasing concentrations of purified Rsp and 4 fmol of the biotin-labelled probe were used in the reactions. The unlabelled probe was added as a specific competitor, and the unlabelled fragment of the *hu* ORF region was added as a nonspecific competitor. (F) Promoter sequence of the *rsp* gene. The start points of the truncated probes are marked by arrows. (G) EMSA assay of Rsp with *rsp* truncated probes. The nonspecific competitor was added to the reactions. The error bars indicate the standard errors of the means of three biological replicates. **P < .01.

Transcriptional autoregulation has been reported for some members of AraC/XylS family and seems to be essential for the transcription of their target genes under stress conditions [53–55]. Our previous work also found that Rbf can specifically bind to the *rbf* promoter to upregulate its own expression [56]. To determine if Rsp has a similar effect on its own transcription, we first detected *rsp* transcription at different growth phases by qRT-PCR, and observed that the transcript level of *rsp* was significantly decreased at the mid-logarithmic phase (Figure 1(A)). Then, we constructed the *lacZ* fusion reporter plasmid pOS*rsp* with the promoter region of *rsp* and detected the β-galactosidase activities in the WT and *rsp* mutant strains. As shown in (Figure 1(B)), the β-galactosidase activity of *rsp* was significantly decreased in the *rsp* mutant strain throughout the growth phases, and the β-galactosidase activities in the WT and *rsp* mutant strains were the highest at mid-logarithmic phase. We examined the mRNA half-lives of p*rsp*-*lacZ* and *rsp* (Figure 1(C)), the results showed although the mRNA half-lives of p*rsp*-*lacZ* and *rsp* are both short, about 20% of p*rsp*-*lacZ* mRNA could survive for more than 1 h. Due to the continuous translation of these RNAs and the stability of LacZ protein, we assumed that the accumulation of LacZ from early exponential phase leads to the high β-galactosidase activity at mid-logarithmic phase.

To test whether the transcription of *rsp* is under the direct control of Rsp, we purified the recombinant Rsp-His_6_ (Figure 1(D)) and employed the 319-bp biotin-labelled putative promoter fragment to perform electrophoretic mobility shift assay (EMSA). The result showed that Rsp could specifically bind to its own promoter region, and the shifted band disappeared when an approximately 200-fold excess of unlabelled *rsp* promoter fragment was added, but not influenced by the addition of 300-fold excess of unlabelled coding sequence fragment of *hu* (Figure 1(E)). Then, to identify the Rsp binding region on its own promoter, we designed the truncated probes of *rsp* (Figure 1(F)). Binding of Rsp was completely inhibited when the probe length was truncated from 139 to 89 bp (Figure 1(G)), suggesting that the Rsp binding sites may located within the 50-bp region. Collectively, these data indicated that Rsp could bind to its own promoter to positively regulate its expression.

### Rsp contributes to haemolytic activity of S. aureus NCTC8325

**Figure 2.**
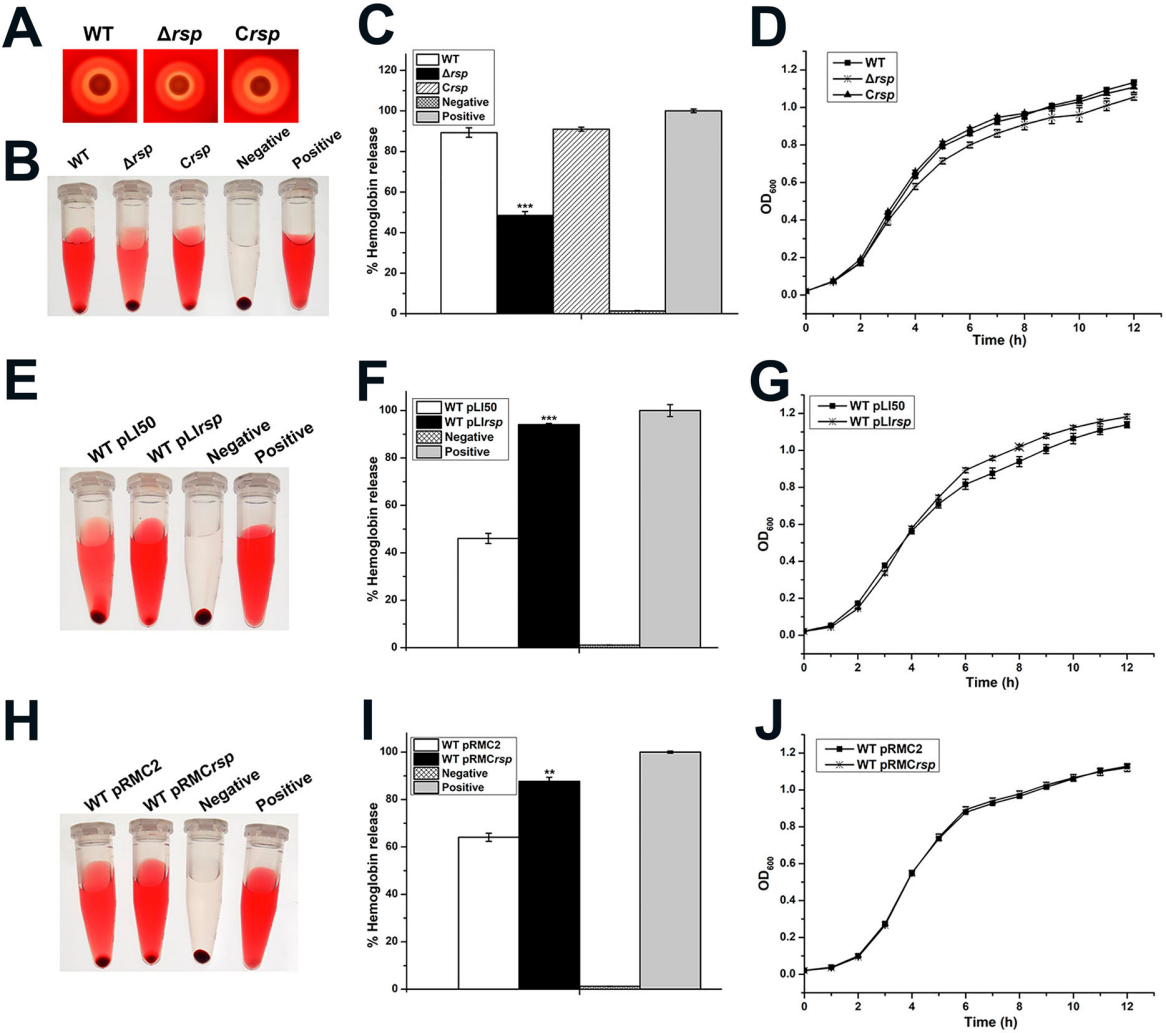
Rsp positively regulates the haemolytic activity of *S. aureus* NCTC8325. (A) Haemolytic activities of the WT, *rsp* mutant, and *rsp* chromosomal-complemented strains were evaluated on SBA plates. (B) Haemolytic activities of the WT, *rsp* mutant, and *rsp* chromosomal-complemented strains were determined by incubating samples with 3% sheep red blood cells, PBS and ddH_2_O were used as negative control and positive control, respectively. (C) Haemolytic activities of the WT, *rsp* mutant, and *rsp* chromosomal-complemented strains were determined by measuring the absorption of supernatants at 543 nm. (D) Comparison of the growth rates of the WT, *rsp* mutant, and *rsp* chromosomal-complemented strains in TSB medium. (E) Haemolytic activities of the WT strains containing plasmids pLI50 and pLI*rsp* were determined by incubating samples with 3% sheep red blood cells. (F) Haemolytic activities of the WT strains containing plasmids pLI50 and pLI*rsp* were determined by measuring the absorption of supernatants at 543 nm. (G) Comparison of the growth rates of the WT strains containing plasmids pLI50 and pLI*rsp* in TSB medium containing 15 μg/ml Cm. (H) Haemolytic activities of the WT strains containing plasmids pRMC2 and pRMC*rsp* were determined by incubating samples with 3% sheep red blood cells. (I) Haemolytic activities of the WT strains containing plasmids pRMC2 and pRMC*rsp* were determined by measuring the absorption of supernatants at 543 nm. (J) Comparison of the growth rates of the WT strains containing plasmids pRMC2 and pRMC*rsp* in TSB medium containing 15 μg/ml Cm and 100 ng/ml ATC. The error bars indicate the standard errors of the means of three biological replicates. **P < .01, ***P < .001.

Rsp has been shown to be involved in the positive regulation of haemolysis in staphylococci [12,13]. To further investigate whether Rsp modulates the haemolysis of *S. aureus* NCTC8325, the haemolytic activities of the WT strain, *rsp* mutant, and *rsp* chromosomal-complemented strains were compared using qualitative and quantitative methods. Qualitative evaluation of haemolytic activity was performed on sheep blood agar plates. As shown in Figure 2(A), the haemolytic activity of the *rsp* mutant strain was much weaker than the WT strain after 24 h of incubation. Quantitative test was performed with the sheep red blood cells, and the percentage of haemolytic activity was calculated relative to the positive control (100% haemolytic activity) by measuring the optical density at 543 nm. The *rsp* mutant strain displayed significantly decreased haemolytic activity compared with the WT strain after incubation for 35 min at 37°C (Figure 2(B,C)). These changes could be restored by the *rsp* chromosomal-complemented strain. In addition, we compared the growth rates of the WT, *rsp* mutant, and *rsp* chromosomal-complemented strains, and found no remarkable difference (Figure 2(D)).

To confirm the regulatory role of Rsp in haemolysis, we overexpressed *rsp* in the WT strain by using a constitutive expression plasmid pLI50 and an ATC inducible expression plasmid pRMC2, and measured the haemolytic activity with the sheep red blood cells. As shown in Figure 2(E,F), the WT strain containing pLI*rsp* exhibited significantly increased haemolytic activity compared with the WT strain containing pLI50 after incubation for 15 min at 37°C. Similarly, the haemolytic activity of the WT strain containing pRMC*rsp* was significantly increased compared with the WT strain containing pRMC2 induced by 100 ng/ml ATC after incubation for 25 min at 37°C (Figure 2(H,I)). Meanwhile, we compared the growth curves of the overexpression strains with their corresponding WT strains, the results showed no significant difference (Figure 2(G,J)). Taken together, our data suggested that the haemolytic activity of *S. aureus* NCTC8325 was modulated by Rsp.

### Rsp positively regulates the transcription of virulence genes

**Figure 3.**
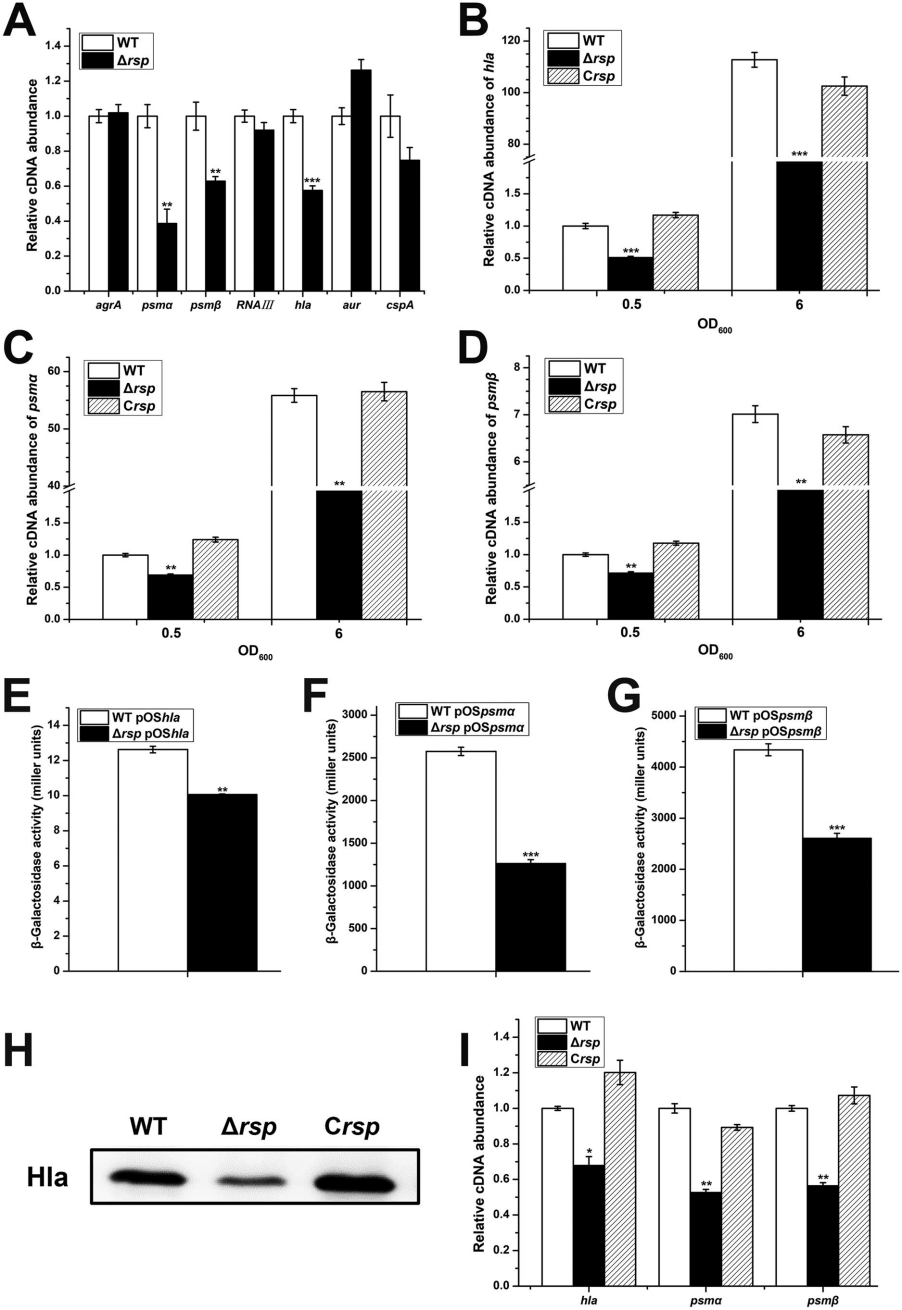
Rsp positively regulates the transcription of virulence genes. (A) The transcript levels of 7 virulence-related genes were detected by qRT-PCR in the WT and *rs*p mutant strains. The transcript levels of *hla* (B), *psmα* (C), and *psmβ* (D) in the WT, *rs*p mutant, and *rs*p chromosomal-complemented strains at different growth phases. The β-galactosidase activities of *hla* (E), *psmα* (F), and *psmβ* (G) promoter in the WT and *rs*p mutant strains. Cells were collected at early stationary phase, and the β-galactosidase activity was detected with ONPG. (H) Hla protein levels of the WT, *rs*p mutant, and *rs*p chromosomal-complemented strains were measured by Western blot. (I) The transcript levels of *hla*, *psmα*, and *psmβ* in the WT, *rs*p mutant, and *rs*p chromosomal-complemented strains in the process of haemolysis. Cells were collected after incubated with 3% sheep red blood cells for 30 min, and the mRNA levels were analysed by qRT-PCR. The error bars indicate the standard errors of the means of three biological replicates. *P < .05, **P < .01, ***P < .001.

The data presented above clearly demonstrated that Rsp is involved in the control of haemolysis in *S. aureus* NCTC8325. Many virulence determinants participated in pathogenesis of *S. aureus* infections can influence the haemolysis. To determine whether the expression of virulence factors was altered in the *rsp* mutant, we performed qRT-PCR to examine the mRNA levels of 7 potential target genes with RNA isolated from the WT and *rsp* mutant strains (Figure 3(A)). The transcript levels of *hla*, *psmα*, and *psmβ* were significantly decreased in the *rsp* mutant strain. Notably, the transcript levels of *agrA* and RNAIII were not altered in the *rsp* mutant strain, implying that the transcriptional changes of *hla* and *psm* is *agr*-independent. To verify the transcriptional control by Rsp, we examined the mRNA levels of *hla*, *psmα*, and *psmβ* at different growth phases. The qRT-PCR results showed that the transcript levels of *hla*, *psmα*, and *psmβ* were significantly decreased in the *rsp* mutant strain at early exponential and stationary phases compared with those of the WT strain, and these effects could be fully restored by chromosomal complementation (Figure 3(B–D)).

To further confirm the positive effect of Rsp on the expression of *hla*, *psmα*, and *psmβ*, the β-galactosidase assay was performed. The *lacZ* fusion reporter plasmids pOS*hla,* pOS*psmα*, and pOS*psmβ* were constructed and transformed into the WT and *rsp* mutant strains, respectively. The results showed that the β-galactosidase activities in the *rsp* mutant strain were significantly lower than those in the WT strain (Figure 3(E–G)), which are consistent with the results of qRT-PCR. Western blot analysis indicated that the production level of Hla was also significantly decreased in the *rsp* mutant strain (Figure 3(H)). To determine the pivotal role of Hla and PSMs in haemolysis, the bacterial cells were collected after incubated with sheep red blood cells, and the mRNA levels were analysed by qRT-PCR. The transcript levels of *hla*, *psmα*, and *psmβ* were decreased significantly in the *rsp* mutant strain (Figure 3(I)). These data suggested that the decrease of haemolytic activity of the *rsp* mutant strain is mainly due to the decreased expression levels of *hla* and *psm*.

### Rsp modulates the haemolytic activity of S. aureus and the transcription of virulence genes in an agr-independent manner

**Figure 4.**
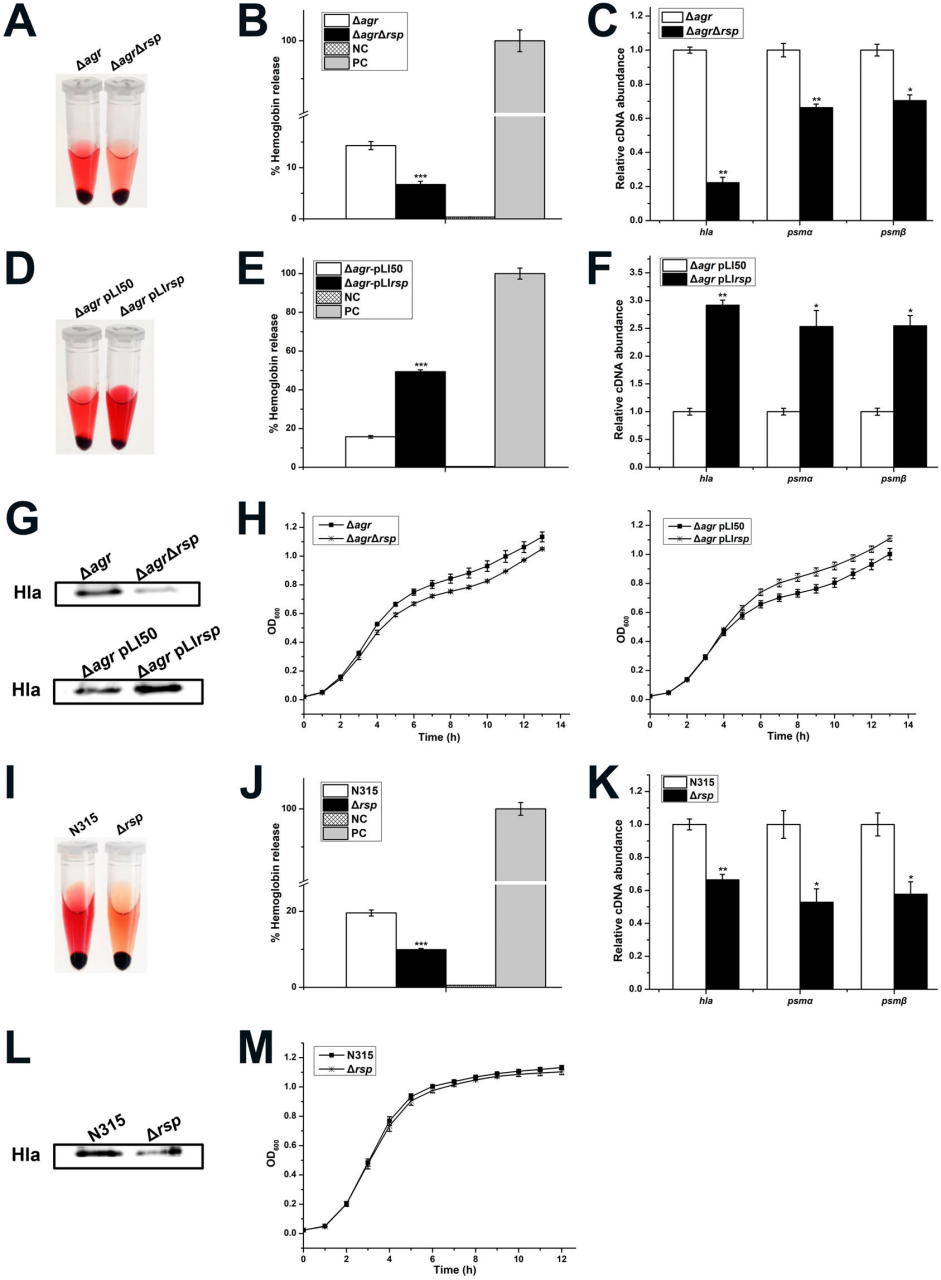
Rsp modulates the haemolytic activity of *S. aureus* and the transcription of virulence genes in an *agr*-independent manner. (A) Haemolytic activities of the *agr* mutant and *agr rsp* double mutant strains were determined by incubating samples with 15% sheep red blood cells, PBS and ddH_2_O were used as negative control and positive control, respectively. (B) Haemolytic activities of the *agr* mutant and *agr rsp* double mutant strains were determined by measuring the absorption of supernatants at 543 nm. (C) The transcript levels of *hla*, *psmα*, and *psmβ* in the *agr* mutant and *agr rsp* double mutant strains. (D) Haemolytic activities of the *agr* mutant strains with the empty plasmid pLI50 and *rsp* overexpressed plasmid pLI*rsp* were determined by incubating samples with 15% sheep red blood cells. (E) Haemolytic activities of the *agr* mutant strains with the empty plasmid pLI50 and *rsp* overexpressed plasmid pLI*rsp* were determined by measuring the absorption of supernatants at 543 nm. (F) The transcript levels of *hla*, *psmα*, and *psmβ* in the *agr* mutant strains with the empty plasmid pLI50 and *rsp* overexpressed plasmid pLI*rsp*. (G) Western blot analysis of Hla. The protein levels of Hla in the *agr* mutant and *agr rsp* double mutant strains as shown in the upper panel. The lower panel shows the protein levels of Hla in the *agr* mutant strains with the empty plasmid pLI50 and *rsp* overexpressed plasmid pLI*rsp*. (H) Comparison of the growth rates of the *agr*-deficient strains. (I) Haemolytic activities of the N315 and *rsp* mutant strains were determined by incubating samples with 15% sheep red blood cells. (J) Haemolytic activities of the N315 and *rsp* mutant strains were determined by measuring the absorption of supernatants at 543 nm. (K) The transcript levels of *hla*, *psmα*, and *psmβ* in the N315 and *rsp* mutant strains. (L) Hla protein levels of the N315 and *rs*p mutant strains were measured by Western blot. (M) Comparison of the growth rates of the N315 and *rsp* mutant strains. The error bars indicate the standard errors of the means of three biological replicates. *P < .05, **P < .01, ***P < .001.

The *agr* has been recognized as a global regulator involved in the regulation of virulence factors [21] and has been proposed to be positively regulated by Rsp [12]. However, as mentioned above, we did not observe the transcriptional change of *agrA* in the *rsp* mutant strain (Figure 3(A)). We further detected the transcript levels of *agrA* at different growth phases, and no difference was found between the WT and *rsp* mutant strains (data not shown). Thus, it is reasonable to assume that Rsp modulates the haemolysis and the expression of virulence genes independent of *agr*. To investigate whether the regulatory effects described here exist in *S. aureus* NCTC8325, we constructed the *agr* mutant and *agr rsp* double mutant strains, and measured the haemolytic activity with the sheep red blood cells. As shown in Figure 4(A,B), the haemolytic activity of the *agr rsp* double mutant strain was significantly decreased compared with the *agr* mutant strain after incubation for 1.5 h at 37°C. We also performed overexpression of the *agr* mutant with the overexpressed plasmid pLI*rsp*. The results showed that transformation of the *agr* mutant with pLI*rsp* increased the haemolytic activity (Figure 4(D,E)). In addition, the growth rates of these strains showed no significant difference (Figure 4(H)).

To confirm if the changes of haemolytic activity were associated with the expression of virulence genes, we examined the transcript levels of *hla*, *psmα*, and *psmβ*. In the *agr rsp* double mutant strain, the expression of *hla*, *psmα*, and *psmβ* were significantly decreased (Figure 4(C)). Introduction of pLI*rsp* into the *agr* mutant increased the transcript levels of *hla*, *psmα*, and *psmβ* (Figure 4(F)). Moreover, we measured the Hla production in these strains by Western blot assay. The results showed that the production level of Hla in the *agr rsp* double mutant strain was much lower than the *agr* mutant strain and transformation of the *agr* mutant with pLI*rsp* displayed a distinctly higher production level of Hla than that in the *agr* mutant with plasmid pLI50 (Figure 4(G)). These results indicated that Rsp modulates the haemolytic activity of *S. aureus* and the transcription of virulence genes in an *agr*-independent manner.

Since Rsp has been reported to regulate the expression of virulence genes in CA-MRSA strains MW2 and BD02-25 via an *agr*-dependent pattern, we were interested in whether the *agr*-independent regulatory pathway mediated by Rsp is specific to HA *S. aureus*. We constructed the *rsp* mutant in HA *S. aureus* strains N315 and Newman and performed haemolytic activity assay. The results showed that the haemolytic activities of the *rsp* mutant strains were significantly decreased compared with those of the WT strains (Figure 4(I,J) and Figure S1(A,B)). Similar with *S. aureus* NCTC8325, no significant difference was observed in the growth rates of the WT and *rsp* mutant strains (Figure 4(M) and Figure S1(E)). We then examined the transcript levels of virulence genes in these strains and the results indicated that the expression of *hla*, *psmα*, and *psmβ* were significantly decreased in the *rsp* mutant strains (Figure 4(K) and Figure S1(C)). Western blot analysis also showed that the *rsp* mutant strains exhibited decreased production of Hla (Figure 4(L) and Figure S1(D)). Additionally, the expression of *agrA* was significantly decreased in the *rsp* mutant strain of *S. aureus* Newman (Figure S1(C)), suggesting that Rsp regulates the transcription of virulence genes in this strain might through an *agr*-dependent manner. Taken together, these results corroborated the regulatory effects of Rsp on the transcription of virulence genes and also proved that they are variable in different *S. aureus* strains.

### Rsp directly binds to the promoter regions of hla, psmα, and psmβ

**Figure 5.**
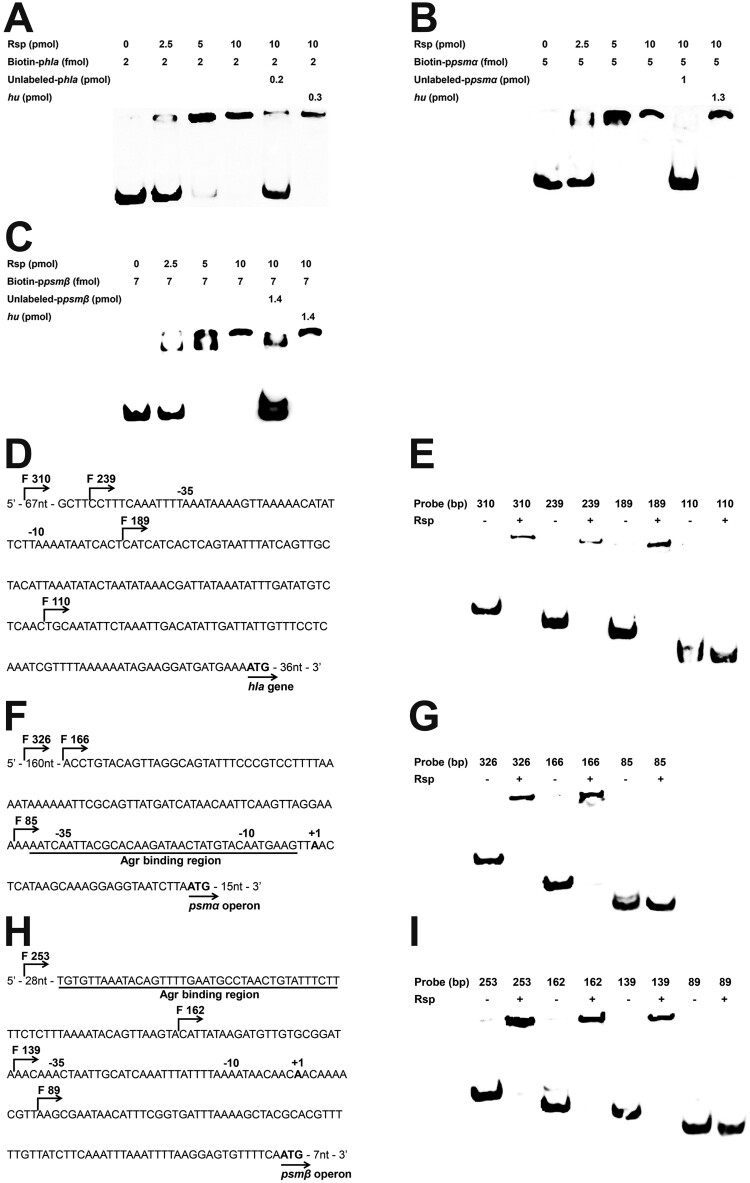
Rsp specifically binds to the *hla*, *psmα*, and *psmβ* promoter regions. EMSA assay of the purified Rsp with the biotin-labelled DNA fragments of *hla* (A), *psmα* (B), or *psmβ* (C). Increasing concentrations of purified Rsp and 2 fmol (*hla*), 4 fmol (*psmα*), or 7 fmol (*psmβ*) of the biotin-labelled probe were used in the reactions. The unlabelled probes were added as a specific competitors, and the unlabelled fragment of *hu* ORF region was added as a nonspecific competitor. (D) Promoter sequence of the *hla* gene. The start points of the truncated probes are marked by arrows. (E) EMSA assay of Rsp with *hla* truncated probes. The nonspecific competitor was added to the reactions. (F) Promoter sequence of the *psmα* operon. The start points of the truncated probes are marked by arrows. The AgrA binding region is underlined in black. (G) EMSA assay of Rsp with *psmα* truncated probes. The nonspecific competitor was added to the reactions. (H) Promoter sequence of the *psmβ* operon. The start points of the truncated probes are marked by arrows. The AgrA binding region is underlined in black. (I) EMSA assay of Rsp with *psmβ* truncated probes. The nonspecific competitor was added to the reactions.

To determine whether *hla*, *psmα*, and *psmβ* are under the direct control of Rsp, we performed EMSA assay. The result showed that Rsp can retard the mobility of the *hla* promoter in a dose-dependent manner (Figure 5(A)). This binding can be disrupted with an approximately 100-fold of unlabelled *hla* promoter fragment, while a 150-fold excess of unlabelled coding sequence fragment of *hu* did not have the same effect. We also employed the *psmα* and *psmβ* promoter regions upstream of the initiation codon to perform an EMSA, and similar binding patterns were observed with the *psmα* and *psmβ* promoters (Figure 5(B,C)). To further identify the Rsp binding regions, EMSA was performed using the truncated probes of *hla*, *psmα*, and *psmβ* (Figure 5(D,F,H)). Concerning *hla*, the binding of Rsp was abolished when the probe length was truncated from 189 to 110 bp (Figure 5(E)). For *psmα*, the shifted bind disappeared when the probe length was truncated from 166 to 85 bp (Figure 5(G)). For *psmβ*, when the probe length was truncated from 139 to 89 bp, the binding was completely inhibited (Figure 5(I)). Additionally, EMSAs with truncated probes of *psmα* and *psmβ* showed that the Rsp binding regions were located upstream and downstream of the AgrA binding sites, respectively [21]. These results strongly suggested that Rsp can directly bind to the promoter regions of *hla*, *psmα*, and *psmβ* to upregulate their expression. Interestingly, no conserved sequence has been found in the Rsp binding regions of *rsp*, *hla*, *psmα*, and *psmβ* promoters through sequence alignment.

### Rsp contributes to virulence of S. aureus in a subcutaneous abscess model of mice

**Figure 6.**
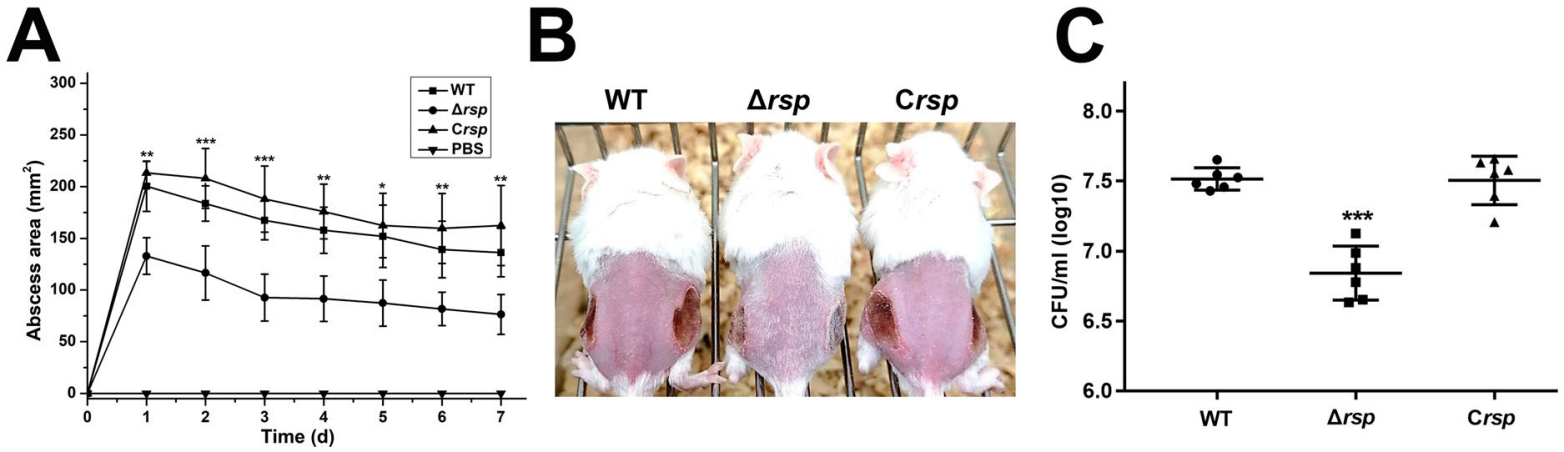
Rsp contributes to virulence of *S. aureus* in a subcutaneous abscess model of mice. The mice were inoculated with 50 μl PBS containing 5 × 10^7^ CFU of the WT, *rsp* mutant, and *rsp* chromosomal-complemented strains, or PBS alone as control, in both flanks of the back by subcutaneous injection. (A) (*n* = 6–8) Abscess area was measured daily with a caliper. (B) The photographic images of representative abscesses in mice 7 days after infection. (C) (*n* = 6) CFU recovered from each abscess harvested 7 days after infection was determined by serial dilution and plated onto TSA plates. The error bars indicate the standard errors of the means of three biological replicates. *P < .05, **P < .01, ***P < .001.

Hla and PSMs are well-characterized toxins, and previous studies have indicated that they play a significant role in *S. aureus* skin and soft-tissue infection [28,29]. To investigate the contribution of Rsp to the virulence of *S. aureus,* the mouse subcutaneous abscess model was used. Mice were administered 50 μl of PBS containing 5 × 10^7^ live *S. aureus*, and the area of abscesses was measured daily after infection. As shown in Figure 6(A,B), the ability of *rsp* mutant strain to cause skin abscesses in mice was significantly decreased compared with the WT and *rsp* chromosomal-complemented strains. The bacterial recovery from the skin abscesses was also significantly decreased in mice infected with the *rsp* mutant strain, and the effect could be fully restored by chromosomal complementation (Figure 6(C)). These results indicated that Rsp can positively regulate the expression of virulence genes to affect the pathogenicity of *S. aureus*.

### Rsp has no influence on the virulence of S. aureus Δagr strain in a subcutaneous abscess model of mice

**Figure 7.**
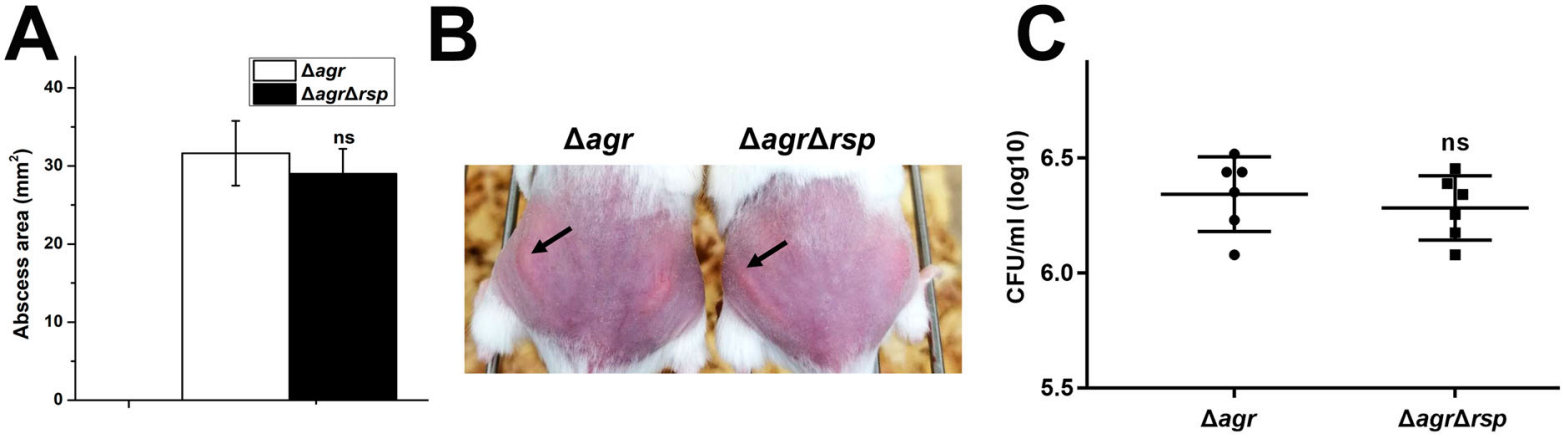
Virulence of the *agr* mutant and *agr rsp* double mutant strains in a subcutaneous abscess model of mice. The mice were inoculated with 50 μl PBS containing 5 × 10^7^ CFU of the *agr* mutant and *agr rsp* double mutant strains, or PBS alone as control, in both flanks of the back by subcutaneous injection. (A) (*n* = 6) Abscess area was measured 4 days after infection. (B) The photographic images of representative abscesses in mice 4 days after infection. Arrowheads indicate the abscesses of mice. (C) (*n* = 6) CFU recovered from each abscess harvested 4 days after infection was determined by serial dilution and plated onto TSA plates. The error bars indicate the standard errors of the means of three biological replicates. NS, not significant (*P* > .05).

Since Rsp can directly regulate the transcription of virulence genes, the mouse subcutaneous abscess model was further used to assess the virulence of the *agr* mutant and *agr rsp* double mutant strains. As shown in Figure 7(B), abscesses without dermonecrosis were present in mice infected with the *agr* mutant strain, which is consistent with previous observation [57]. The area of abscesses (Figure 7(A)) and the bacterial loads (Figure 7(C)) were similar in mice administered by 5 × 10^7^ CFU of the *agr* mutant and *agr rsp* double mutant strains. In addition, transformation of the *agr* mutant with pLI*rsp* also did not affect its pathogenicity, as measured by abscess formation abilities and bacterial loads (data not shown). These data suggested that Rsp has no influence on the virulence of the *agr* mutant strain in mouse skin infection model. We figured that the dramatic impact of Agr on the expression of virulence genes and pathogenicity of *S. aureus* might weaken the difference in virulence between these strains.

## Discussion

The success of *S. aureus* as an opportunistic human pathogen is typically attributed to its ability to cause a diverse array of infections and syndromes, which requires the coordination of various virulence factors and global regulators. The studies on the global virulence regulators, including Agr system, Sar family, and Sae two-component system have shown that the virulence regulation network of this bacterium is highly complex [58,59]. Thus, much remains to be elucidated to fully understand the molecular mechanisms of the virulence and pathogenesis of *S. aureus*. In this study we attempted to further investigate the physiological roles of Rsp in *S. aureus* NCTC8325.

Rsp belongs to the AraC/XylS family transcriptional regulators, which are involved in the regulation of carbon source utilization, stress responses, pathogenesis and general metabolism, either as a transcriptional activators or a transcriptional repressors [6]. The results of β-galactosidase activity assay and EMSA revealed that Rsp can specifically bind to the *rsp* putative promoter region to upregulate its own expression. Moreover, except HptR, all of the AraC/XylS family members of *S. aureus* have an atypical features with the conserved 99 amino acid region containing two helix-turn-helix (HTH) DNA binding motifs at the N-terminal and the divergent C-terminal region, which seems to be involved in environmental signals recognition and protein multimerization [6]. Recently, Dasa et al. [13] found that the transcript levels of *rsp* and *rsp* regulon were rapidly up-regulated in response to hydrogen peroxide, which is produced by neutrophils. Thus, it is reasonable to assume that Rsp can be modulated by interacting with effectors that accumulate in the infection sites and then facilitate *S. aureus* to adapt to the specific, often hostile environment by altering the expression of large pools of genes.

Previous studies have demonstrated that Rsp is a positive regulator of *S. aureu*s haemolysis [12,13]. In this study, we also found that loss of *rsp* can significantly reduce haemolytic activity in *S. aureus* NCTC8325. The function of Rsp in haemolysis was further determined by introducing the pLI*rsp* and pRMC*rsp* plasmids in which *rsp* was highly overexpressed into the WT strain. Many secreted toxins involved in pathogenesis of *S. aureus* infections can influence the haemolysis. The results of qRT-PCR, β-galactosidase activity assay, western blot, and EMSA evidenced that Rsp can upregulate the expression of *hla*, *psmα*, and *psmβ* by directly binding to their promoter regions.

It has been reported that the expression of *hla* and *psm* is tightly regulated by the *agr* locus [21,36], and that Rsp can regulate the expression of virulence genes via an *agr*-dependent manner [12]. However, there were no dramatic differences in the expression of *agrA* and RNAIII between the WT and *rsp* mutant strains in our study, suggesting that Rsp might regulate the expression of virulence genes in an *agr*-independent manner in *S. aureus* NCTC8325. We examined the haemolysis of the *agr*-mutant strains, and found that Rsp can still promote the haemolysis of *S. aureus* in the absence of *agr*. We also checked the transcript levels of *hla* and *psm* and the production level of Hla in the *agr*-mutant strains, and the results showed the same tendency. These results suggested that Rsp can modulate the haemolytic activity of *S. aureus* and the transcription of virulence genes independent of *agr*. Meanwhile, we constructed the *rsp* mutant in HA *S. aureus* strains N315 and Newman to further investigate whether the *agr*-independent Rsp regulatory pathway is HA *S. aureus*-specific. The results of haemolysis, qRT-PCR, and western blot corroborated the regulatory effects of Rsp on the transcription of virulence genes. Mutation of *rsp* in *S. aureus* N315 displayed decreased transcription of virulence genes independent of *agr*. However, in *S. aureus* Newman, mutation of *rsp* was correlated with the decreased expression of *agr*, suggesting that Rsp regulates the transcription of virulence genes in this strain might through an *agr*-dependent manner. These results suggested that the regulatory effects of Rsp on the transcription of virulence genes are variable in different *S. aureus* strains. A previous report has shown that mutation of *rsp* decreased the haemolytic activity and the expression of virulence genes in *S. aureus* USA300 LAC, but had no impact on the expression of *agr* [13], further indicating that neither the *agr*-dependent nor *agr*-independent regulatory pathways mediated by Rsp are strain specific.

Since our data indicated that Rsp contributes to the haemolysis, a hallmark of *S. aureus* virulence, and that Rsp positively regulates the expression of Hla and PSMs, which are essential for *S. aureus* to cause diseases in vivo [28,29,60], we further investigated the impact of *rsp* on virulence using mouse subcutaneous abscess model. Unlike systemic bacteraemia model, which evaluates the ability of bacteria to survive, disseminate and proliferate, the subcutaneous abscess infection does not require dissemination, in which bacterial growth is restricted by the infiltration of polymorphonuclear leukocytes and nutrient limitations [61]. The *rsp* mutant strain displayed decreased virulence compared with the WT strain, as characterized by a defect in abscess formation and a blunt in bacterial recovery from the abscesses. This finding is also supported by the previous studies of *S. aureus* infections [12,13]. Furthermore, due to the abscess formation ability of the *agr* mutant was almost abolished, we failed to observe significant difference in virulence between the *agr* mutant and the *agr rsp* double mutant strains. Whether Rsp modulates the virulence of *S. aureus* in the absence of *agr* remains to be investigated by other mouse infection models.

In addition to the virulence regulation, Rsp also has been reported to play an important role in the regulation of biofilm formation in *S. aureus* [11,12]. In this study, we found that the *rsp* mutants resulted in increased biofilm formation ability compared with those of the WT strains (Figure S2(A–C)). Furthermore, compared with the *agr* mutant strain, the *agr rsp* double mutant strain displayed increased biofilm formation both in the NCTC8325 and N315 backgrounds (Figure S2(A,B)), indicating that Rsp can modulate biofilm formation in an *agr*-independent manner. Considering the significance of PSMs in biofilm formation [62], we assumed that the increased biofilm formation in the *rsp* mutant strain might partially associated with the decreased expression of *psm*.

In conclusion, this study has revealed that Rsp can directly bind to its own promoter region for autoregulation, and that Rsp can positively regulate the expression of virulence genes by directly binding to their promoter regions in an *agr*-independent manner. The contribution of Rsp to the pathogenicity of *S. aureus* was confirmed by using a mouse subcutaneous abscess model. These findings provide new insights into the regulatory mechanisms of virulence gene expression and *S. aureus* pathogenesis.

## Supplementary Material

Supplemental Material
