# Supplementary figures and images for: Rsp promotes the transcription of virulence factors in an agr-independent manner in Staphylococcus aureus
Source: Emerg Microbes Infect. 2020 Apr 24;9(1):796–812. doi: 10.1080/22221751.2020.1752116 (PMC7241556; doi:10.1080/22221751.2020.1752116)

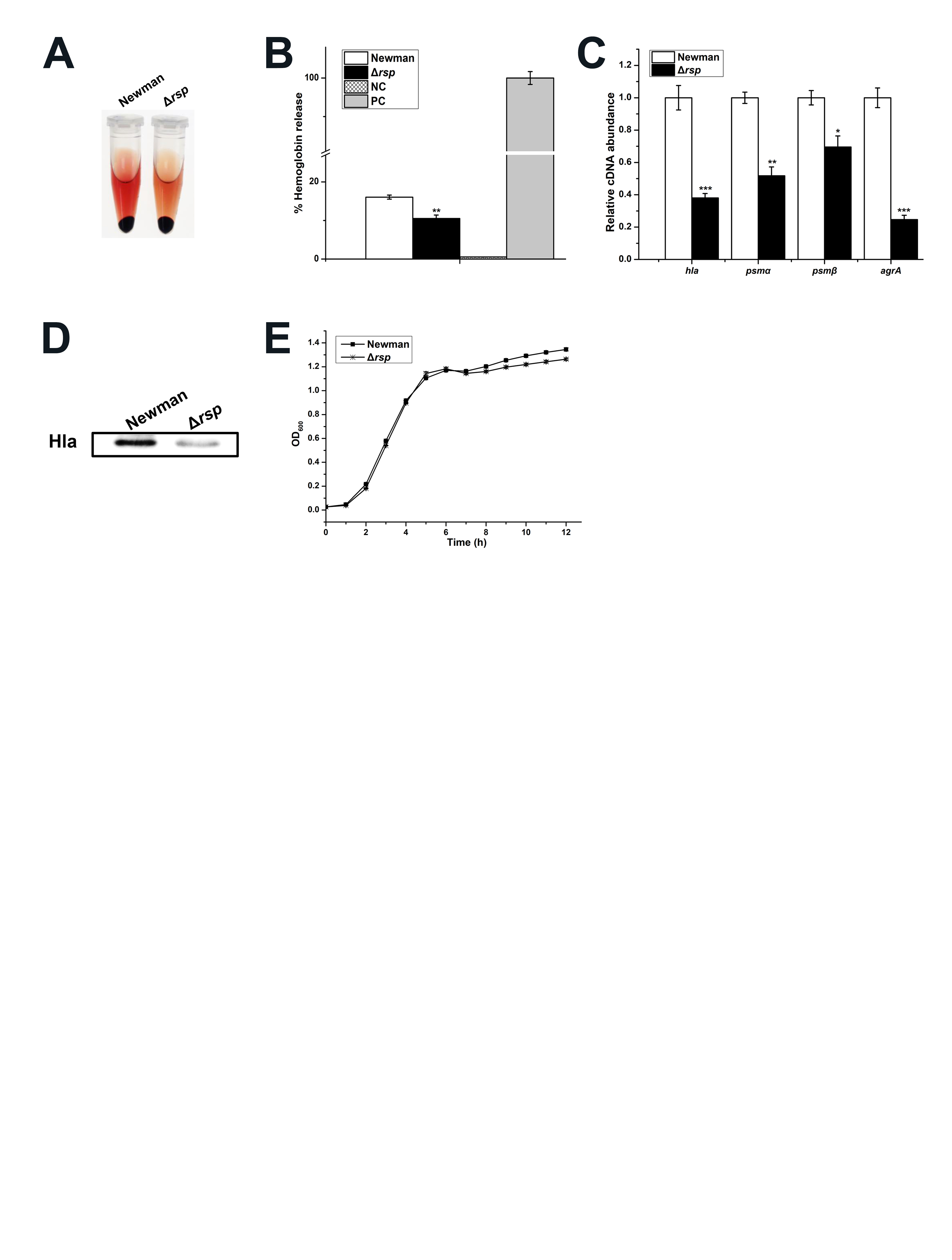

Supplement: Supplemental Material [file TEMI_A_1752116_SM5645.zip › Sun_Figure S1.jpg]

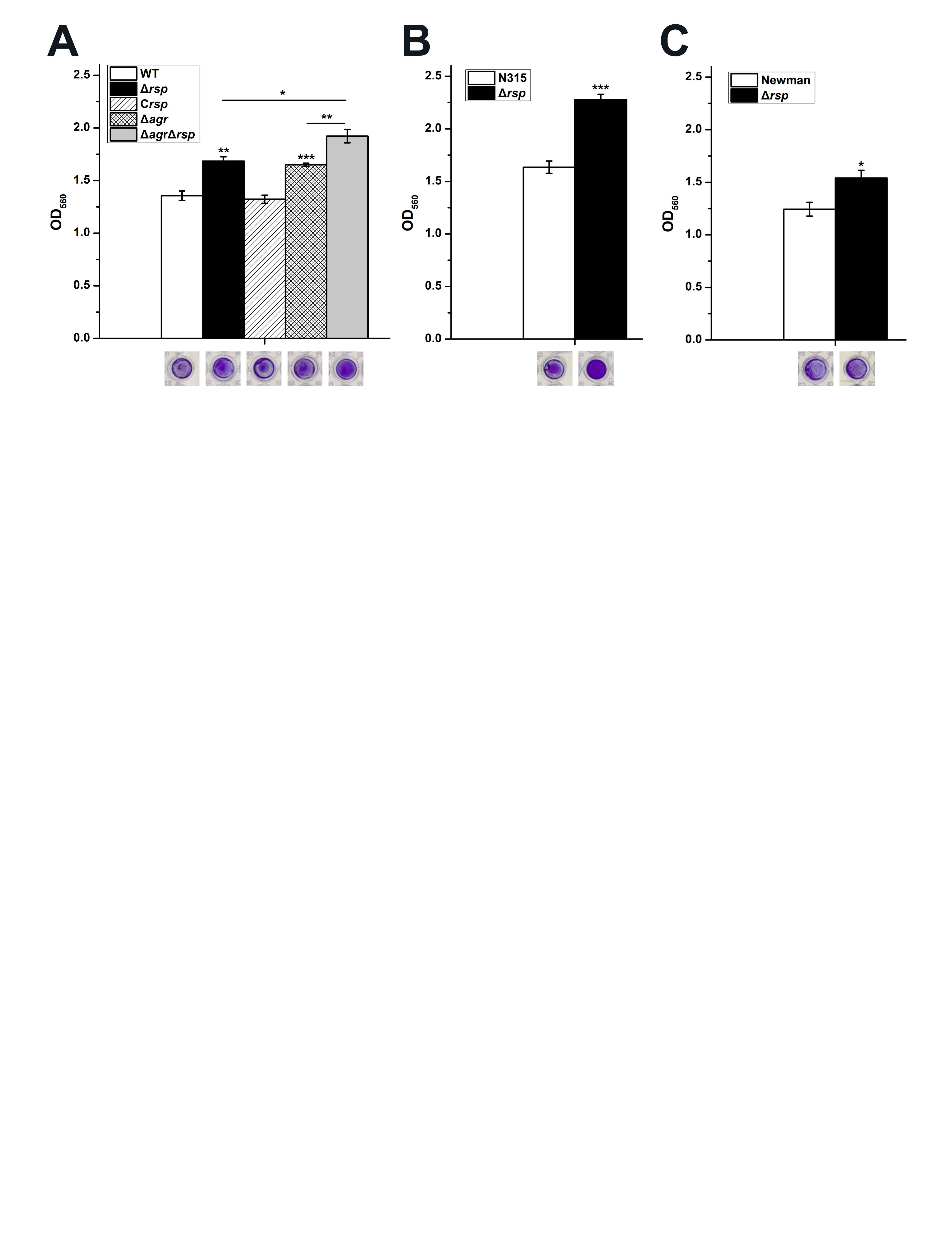

Supplement: Supplemental Material [file TEMI_A_1752116_SM5645.zip › Sun_Figure S2.jpg]
